# Simultaneous Nitrogen Removal and Plant Growth Promotion Using Salt-‍tolerant Denitrifying Bacteria in Agricultural Wastewater

**DOI:** 10.1264/jsme2.ME22025

**Published:** 2022-09-17

**Authors:** Huanhuan Zhang, Weishou Shen, Changyi Ma, Shanshan Li, Jie Chen, Xinfei Mou, Wenwen Cheng, Peng Lei, Hong Xu, Nan Gao, Keishi Senoo

**Affiliations:** 1 School of Biotechnology and Pharmaceutical Engineering, Nanjing Tech University, Nanjing 211816, China; 2 Jiangsu Key Laboratory of Atmospheric Environment Monitoring and Pollution Control, Collaborative Innovation Center of Atmospheric Environment and Equipment Technology, and School of Environmental Science and Engineering, Nanjing University of Information Science and Technology, Nanjing 210044, China; 3 School of 2011, Nanjing Tech University, Nanjing 211816, China; 4 School of Food Science and Light Industry, Nanjing Tech University, Nanjing 211816, China; 5 Department of Applied Biological Chemistry, Graduate School of Agricultural and Life Sciences, The University of Tokyo, Tokyo 113–8657, Japan; 6 Collaborative Research Institute for Innovative Microbiology, The University of Tokyo, Tokyo 113–8657, Japan

**Keywords:** denitrifying bacteria, salinity resistance, plant growth-promoting rhizobacteria (PGPR), N removal

## Abstract

Excess nitrate (NO_3_^–^) and nitrite (NO_2_^–^) in surface waters adversely affect human and environmental health. Bacteria with the ability to remove nitrogen (N) have been isolated to reduce water pollution caused by the excessive use of N fertilizer. To obtain plant growth-promoting rhizobacteria (PGPR) with salt tolerance and NO_3_^–^-N removal abilities, bacterial strains were isolated from plant rhizosphere soils, their plant growth-promoting effects were evaluated using tomato in plate assays, and their NO_3_^–^-N removal abilities were tested under different salinity, initial pH, carbon source, and agriculture wastewater conditions. The results obtained showed that among the seven strains examined, five significantly increased the dry weight of tomato plants. Two strains, *Pseudomonas stutzeri* NRCB010 and *Bacillus velezensis* NRCB026, showed good plant growth-promoting effects, salinity resistance, and NO_3_^–^-N removal abilities. The maximum NO_3_^–^-N removal rates from denitrifying medium were recorded by NRCB010 (90.6%) and NRCB026 (92.0%) at pH 7.0. Higher NO_3_^–^-N removal rates were achieved using glucose or glycerin as the sole carbon source. The total N (TN) removal rates of NRCB010 and NRCB026 were 90.6 and 66.7% in farmland effluents, respectively, and 79.9 and 81.6% in aquaculture water, respectively. These results demonstrate the potential of NRCB010 and NRCB026 in the development of novel biofertilizers and their use in reducing N pollution in water.

Synthetic nitrogen (N) fertilizers are used to maintain crop yield. However, nitrogen loss via agricultural nonpoint sources may lead to eutrophication in aquatic ecosystems (Min and Shi, 2018; Lu *et al.*, 2021) and contribute towards 19–61% of global surface water pollution (Van Drecht *et al.*, 2003). Excess nitrate (NO_3_^–^) and nitrite (NO_2_^–^) in surface waters adversely affect human and environmental health (Yang *et al.*, 2019). To control N loss from agricultural fields, some technologies, such as mechanical side-deep fertilization, have also been reported as useful mitigation techniques (Min *et al.*, 2021). A number of chemical compounds that enhance NO_3_^–^ removal from water body systems have been identified in root exudates (Lu *et al.*, 2021). Previous studies reported the presence of plant growth-promoting rhizobacteria (PGPR) with N removal abilities (Gao *et al.*, 2016; Zhu *et al.*, 2020; Lu *et al.*, 2021). PGPR have been shown to promote plant growth and induce plant resistance against abiotic stress, eliminate soil and water pollutants, and effectively reduce the amount of synthetic N fertilizers and the negative environmental effects of N (Gao *et al.*, 2016; Zhu *et al.*, 2020; Lu *et al.*, 2021). Biological denitrification is an effective method for N removal from wastewater (Fujitani *et al.*, 2013; Lv *et al.*, 2017; Lu *et al.*, 2021; Xu *et al.*, 2021).

Since N in fields is often the limiting nutrient for crops (particularly rice), denitrifiers applied to the field may accelerate the mineralization of biologically available N. Denitrifying bacteria reduce NO_3_^–^ to N_2_O or N_2_, thereby reducing the amount of NO_3_^–^ and NO_2_^–^ in the water. Bacteria with N removal abilities have recently been isolated to reduce water pollution caused by the excessive use of N fertilizers (Fujitani *et al.*, 2013; Xu *et al.*, 2021). The heterotrophic nitrifier *Acinetobacter junii* YB removed 88.9% of NO_3_^–^-N after 24 h of culture (Yang *et al.*, 2015). Furthermore, coastal farmland and aquaculture wastewaters contain high concentrations of N and soluble salt. High salinity levels restrict the denitrification capacity of most denitrifying bacteria (Tang *et al.*, 2014). In high salinity environments (>1%), the growth and metabolism of denitrifying bacteria are generally inhibited due to ion toxicity, hypertonic stress, and oxidative damage (Oliveira *et al.*, 2016). Only a few salt-tolerant denitrifying bacteria have been isolated to date. For example, *Pseudomonas sp.* MSD4 was isolated from a sewage treatment plant and exhibited denitrification ability at 0–10% salinity. Moreover, it removed 62.4% of TN (100‍ ‍mg L^–1^) at 7% salinity and pH 7.0 and 72.2% of TN at 3% salinity and pH 9.0 (Zeng *et al.*, 2020). When cultured under 30 and 70‍ ‍g L^–1^ NaCl conditions, *Panagrobacterium phragneum* F1 removed 23 and 36% of N (Wang *et al.*, 2019). *Bacillus sp.* N31, with halophilic heterotrophic nitrification-aerobic denitrification properties, was isolated from mariculture water. Its NH_4_^+^-N removal rate significantly increased when salinity ranged between 5 and 30‍ ‍g‍ ‍L^–1^, and decreased from 80.17 to 61.23% with changes in salinity from 40 to 50‍ ‍g L^–1^ (Huang *et al.*, 2017). These strains are suitable for removing NO_3_^–^ from high-salt wastewaters, particularly excess N from coastal agricultural water. If denitrifying bacteria with high salt-tolerant denitrification abilities promote plant growth, they may be developed as biofertilizers.

The initial pH and carbon (C) source play important roles in the N removal process. pH is one of the primary external factors that affects the growth and process of denitrification (Saleh-Lakha *et al.*, 2009). The majority of denitrifying bacteria survive and function in neutral and weakly alkaline environments (pH 6.0–9.0) (Ye *et al.*, 2016). The N removal efficiency of denitrifying bacteria slightly decreases when they are exposed to coastal aquaculture wastewater with pH ranging between 7.0 and 10.0 or alkaline-saline farmland watery discharge with pH ≥10.0 (Dendooven *et al.*, 2010; Li *et al.*, 2018). C serves as an energy source and electron donor for heterotrophic denitrifying bacteria, which strongly influences cell growth and the denitrification process (Marchant *et al.*, 2017). Some denitrifying bacteria prefer glucose and resist acid, while others exhibit a preference for fumaric acid and resist alkali (Duan *et al.*, 2015; Yang *et al.*, 2019; Cheng *et al.*, 2020). C sources markedly affect the efficiency of denitrification (Yang *et al.*, 2015). The NO_3_^–^-N removal rate previously reached 90.0% for *Fusarium solani* RADF-77 when glucose was used as the C source, but was only 19.7% when lactose was the C source (Cheng *et al.*, 2020). The NO_3_^–^-N removal rate reached 97.9% for *Pseudomonas stutzeri* XL-2 after 24 h with sodium acetate as the C source (Zhao *et al.*, 2018).

If a PGPR strain with high salt-tolerant denitrification ability is obtained, it has great potential for practical applications in aquaculture wastewater and agricultural non-point source pollution, possibly under saline-alkaline conditions. In the present study, seven strains were isolated from rhizosphere soil with growth-promoting and salt-tolerant N removal capacities. Of these, two isolates were named NRCB010 and NRCB026. N removal performance at varying pH levels using different C sources under saline conditions were tested. The application prospects of these two strains to actual wastewater were also examined.

## Materials and Methods

### Bacterial isolation

Seven bacterial strains were used in the present study. Five bacterial strains, NRCB001, NRCB010, NRCB023, NRCB024, and NRCB025, were previously isolated from rice rhizosphere soil collected in Yixing (31°12′N, 119°52′E) of Jiangsu Province, China (Zhu *et al.*, 2020). NRCB026 was isolated from rice rhizosphere soil collected in Huizhou (23°1′N, 114°5′E) of Guangdong Province, China. NRCB030 was isolated from common reed (*Phragmites communis* [Cav.] Trin. ex Steud.) rhizosphere soil collected in Nanjing (32°4′N; 118°38′E) of Jiangsu Province, China. To isolate NRCB026 and NRCB030, 1‍ ‍g of plant rhizosphere soil was added to 100‍ ‍mL of 0.86% NaCl and incubated at 30°C for 7 days. The soil-NaCl suspension was shaken at 200‍ ‍rpm for 20‍ ‍min, and approximately 80‍ ‍mL of the suspension was then transferred to a culture flask, to which 40‍ ‍mL of 2.1‍ ‍μmol L^–1^ sodium nitrate and 40‍ ‍mL of 1.4‍ ‍μmol L^–1^ sodium succinate were added. The suspension was incubated statically at 30°C for 24 h. One hundred microliters of five serial supernatant dilutions were plated on nitrogen free broth (NBF) and 1/10 modified nutrient broth containing sodium nitrate and sodium succinate (NBNS) (highly peptone 0.5‍ ‍g‍ ‍L^–1^, LAB-Lemco powder [OXOID] 0.3‍ ‍g L^–1^, sodium nitrate 2.5‍ ‍mg L^–1^, and sodium succinate 71‍ ‍mg L^–1^, pH 7.0). After an incubation at 30°C for 2 days, colonies that differed in appearance were isolated and maintained in 30% (w/v) glycerol at –80°C for further characterization.

### Physiological and plant growth-promoting assays

Ammonia production, NO_3_^–^ reduction, starch hydrolysis, and Voges-Proskaue (V-P) tests were performed according to Bergey’s Manual of Systematic Bacteriology (Goodfellow *et al.*, 2012). A Biolog GEN III MicroStation semi-automated bacterial identification system was used to evaluate the microbial utilization of glycerol and sensitivity to pH (BioTek Instrument).

Indole acetic acid (IAA) production was assessed using the method described by Gao *et al.* (2016) with minor modifications. Briefly, strains were precultured in NBNS liquid medium for 12 h. One milliliter of the pre-cultured strain was added to 9‍ ‍mL of R2A liquid medium supplemented with tryptophan (0.2‍ ‍g L^–1^), followed by shaking at 30°C and 200‍ ‍rpm for 48 h. The cultured suspension was centrifuged at 6,000‍ ‍rpm for 10‍ ‍min, and 2‍ ‍mL of the supernatant was mixed with 2‍ ‍mL of Salkowski reagent (1‍ ‍mL of 0.5‍ ‍mol‍ ‍L^–1^ FeCl_3_+50‍ ‍mL 35% HClO_4_) and incubated at 37°C for 1‍ ‍h in the dark. The concentration of IAA was measured at 530‍ ‍nm using a spectrophotometer (722s; Shanghai Instrument Analytical Instrument). IAA concentrations were assessed using an IAA standard curve ranging between 10 and 500‍ ‍μg mL^–1^. Each treatment was performed in triplicate.

Phosphate-solubilizing capacity was measured according to Zhu *et al.* (2020), and Ca_3_(PO_4_) was used as the sole phosphorus source. The production of siderophore and 1-aminocyclopropane-1-carboxyacetate (ACC) was analyzed according to the method described by Zhu *et al.* (2020).

### Vigor of the tomato plant seedling assay

To evaluate plant growth-promoting effects, seven bacteria were inoculated into tomato seeds (Haoweidao; Xingke Seed) on agar plates. In brief, strains were cultured in NBNS liquid medium, centrifuged, and resuspended in sterile 1/10 Murashige and Skoog (MS) salt (MS without organic elements, agar, or sucrose) (Hope Bio-Technology) at pH 5.8. Strain suspensions were adjusted to an OD_600_ of 1.0 and diluted 100 times. Seeds were washed in 2.5% sodium hypochlorite solution for 10‍ ‍min and further washed with sterile distilled water. Surface-sterilized seeds were soaked in a 100-fold diluted strain suspension for 30‍ ‍min, after which the inoculated seeds were placed on 1/10 MS medium (0.6% sucrose and 0.8% agar; Hope Bio-Technology). Seeds were cultured at 24°C for 10 days under a 16-h photoperiod, after which their height, root length, and dry weight were measured. Each treatment was performed in triplicate.

### Evaluation of bacterial tolerance to salinity

To evaluate salinity effects, strains were precultured in LB liquid medium (tryptone 10‍ ‍g‍ ‍L^–1^, yeast extract 5‍ ‍g‍ ‍L^–1^, and NaCl 10‍ ‍g‍ ‍L^–1^) for 12 h. One milliliter of the pre-cultured strain (an OD_600_ of approximately 1.0) was added to 100‍ ‍mL of LB-based liquid medium (tryptone 10‍ ‍g L^–1^ and yeast extract 5‍ ‍g L^–1^) supplemented with six different salinities (0, 0.2, 0.4, 0.6, 0.8, and 1.0‍ ‍mol‍ ‍L^–1^ NaCl), followed by shaking at 30°C and 200‍ ‍rpm for 48 h. OD_600_ was measured using a spectrophotometer. Each treatment was performed in quadruplicate.

### Evaluation of NO_3_^–^ and NO_2_^–^ removal by bacteria under saline conditions

One microliter of the inoculum of each strain with an OD_600_ of approximately 1.0 was added aseptically to 100‍ ‍mL of sterile salted denitrifying medium (DM; KNO_3_ 0.72‍ ‍g L^–1^, K_2_HPO_4_ 1‍ ‍g‍ ‍L^–1^, sodium succinate 2.8‍ ‍g L^–1^, and MgSO_4_·7H_2_O 1‍ ‍g L^–1^) in a 250-mL shaker flask as described by Zeng *et al.* (2020). Potassium nitrate (100‍ ‍mg L^–1^ NO_3_^–^-N) was used as the sole N source and the initial pH of DM medium was adjusted to 6.8. The initial salinities of DM were 0, 0.2, 0.4, 0.6, and 0.8 mol L^–1^ NaCl. Bacteria were cultured at 30°C and 200‍ ‍rpm for 48 h, after which OD_600_ and pH were measured. The suspensions were centrifuged, NO_3_^–^-N in the supernatant was measured by UV spectrophotometry at wavelengths of 220 and 275‍ ‍nm (Lu, 1999), and NO_2_^–^-N in the supernatant was measured using the diazotization coupling spectrophotometric method at a wavelength of 530‍ ‍nm (Lu, 1999). Each treatment was performed in triplicate.

### Bacterial identification using ribosomal and housekeeping genes

Genomic DNA was extracted from NRCB010 and NRCB026 using the FastPure^®^ Bacteria DNA Isolation Mini Kit (Vazyme Biotech) according to the manufacturer’s protocol. The *16S rRNA* gene was amplified using an S1000 Thermal Cycler (Bio-Rad) in 10-μL reaction volumes containing 5‍ ‍μL of 2×Taq Master Mix (Dye Plus), 200‍ ‍ng of template DNA, 1‍ ‍μL of each primer (10‍ ‍μmol L^–1^), and sterile distilled water. Primers and thermal profiles are listed in Table S1 (Polz *et al.*, 1999; Zhu *et al.*, 2020). PCR products were confirmed by 1.0% agarose gel electrophoresis, after which they were purified and sequenced at Suzhou Jinweizhi Biotechnology. The online Basic Local Alignment Search Tool (BLASTN) program was used to search for related sequences with known taxonomic information on the NCBI website (http://www.ncbi.nlm.nih.gov/BLAST) in order to compare the strains. The phylogenetic tree was constructed in MEGA 5.0 using the neighbor-joining method with 1,000 bootstrap replicates.

To identify more intimate inter- and intra-specific relationships for NRCB026, the *DNA gyrase subunit A* gene (*gyrA*) and *DNA gyrase subunit B* gene (*gyrB*) were amplified and sequenced as described above. The *gyrA* and *gyrB* sequences obtained were used for a multilocus sequence ana­lysis (MLSA). The nucleotide sequence alignment of concatenated genes consisted of 2,033 bp (1,197 bp from *gyrA* and 836 bp from *gryB*). The phylogenetic tree was constructed as described above.

### N removal in culture media with different initial pH and C sources

The effects of initial pH and C sources in culture medium on N removal by *P. stutzeri* NRCB010 and *Bacillus velezensis* NRCB026 were evaluated under 0.6 mol L^–1^ NaCl conditions. To test the initial pH effects, the pH values of DM were adjusted to 6.0, 7.0, 8.0, 9.0, and 10.0. To test the C source effects, glucose, glycerin, and sodium acetate were selected as C sources to replace sodium succinate in DM. One milliliter of the strain suspension (an OD_600_ of approximately 1.0, about 6.0×10^9^ CFU for NRCB010 and 2.8×10^9^ CFU for NRCB026) was inoculated into 100‍ ‍mL sterile medium and cultured at 30°C and 200‍ ‍rpm for 72 h. OD_600_, NO_3_^–^-N, and NO_2_^–^-N were measured spectrophotometrically after an incubation for 0, 6, 12, 24, 30, 36, 48, and 72 h. Each treatment was performed in triplicate.

### N removal from agricultural waters

The N removal potentials of NRCB010 and NRCB026 were evaluated in agricultural water samples. Water samples were collected from a fishpond at Nanjing Tech University and a field ditch in Liuhe, Nanjing. In total, 2.5 milliliters of strain suspensions (OD_600_=1.0) were inoculated into 250‍ ‍mL of each water sample, and non-inoculated water was used as the control. Water samples were incubated at 30°C and 200‍ ‍rpm for 72 h. Water samples were sampled at 6, 12, 24, 36, 48, 60, and 72 h to measure TN using the potassium persulfate oxidation-ultraviolet spectrophotometric method (Lu, 1999). Each treatment was performed in triplicate.

### Statistical ana­lysis

IBM SPSS statistics for Windows (Version 26.0, IBM Corp., 2019) was used for statistical ana­lyses. All data were expressed as means±standard errors. Significant differences were calculated by a one-way ANOVA with Duncan’s multiple range test (*P*<0.05).

## Results

### Bacterial isolation and physiological characterization

Seven colonies with different morphologies were isolated and named as follows: NRCB001, NRCB010, NRCB023, NRCB024, NRCB025, NRCB026, and NRCB030. NRCB010, NRCB023, and NRCB025 were positive when subjected to ammonia production, NO_3_^–^ reduction, and starch hydrolysis tests as well as glycerin utilization (Table S2). NRCB026 was also positive when subjected to the NO_3_^–^ reduction test, V-P test, growth at pH 5.0, and glycerin utilization (Table S2).

### Plant growth-promoting effects of isolates

Varying concentrations (1.19–33.33‍ ‍μg mL^–1^) of IAA were produced by the seven isolates, among which NRCB010, NRCB023, and NRCB026 showed higher concentrations than the other strains (Table 1). The seven isolates exhibited phosphate-solubilizing abilities (50–60‍ ‍mg‍ ‍L^–1^), siderophore activities (29.26–52.3%), and ACC deaminase activities.

**Fig. 1.**
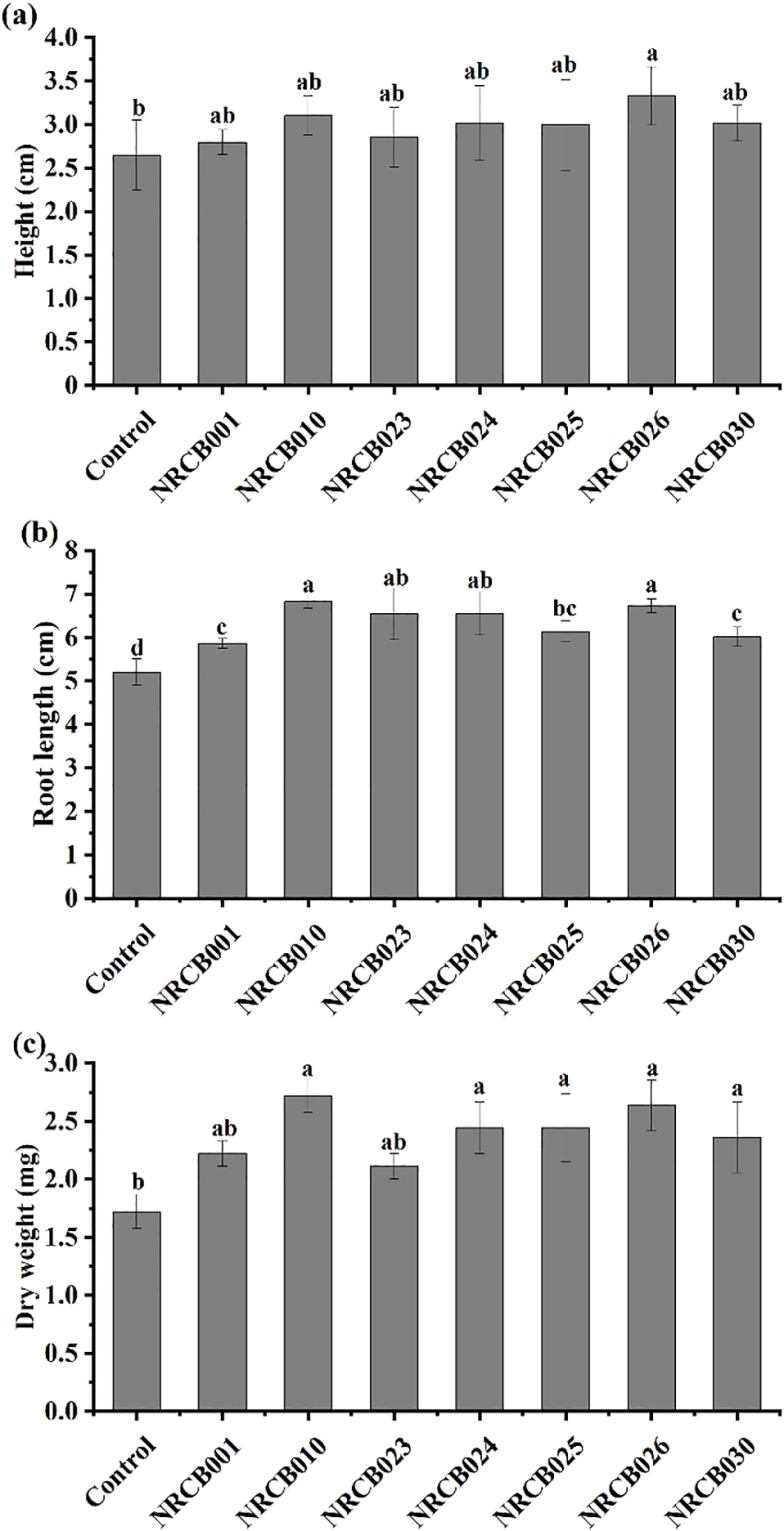
Effects of bacterial inoculations on the height (a), root length (b), and dry weight (c) of tomato seedlings. Data are means±standard errors (*n*=3). The same letter above the bars is not significantly different at *P*<0.05 by Duncan’s post-hoc test.

In comparisons with the non-inoculated control, the height and root length of tomato seedlings significantly increased after inoculations with the seven isolates (*P*<0.05) (Fig. 1a and b). The dry weight of tomato seedlings also significantly increased after inoculations with five strains (*P*<0.05) (Fig. 1c). Dry weights increased by 58.1 and 53.2% after inoculations with NRCB010 and NRCB026 (*P*<0.05), respectively. NRCB010 and NRCB026 exerted stronger plant growth-promoting effects than the other strains.

### Salinity tolerance of isolates

After a 48-h incubation in LB-based liquid media containing 0.2 to 0.6 mol L^–1^ NaCl, six isolates, except for NRCB001, showed good growth (OD_600_ higher than 1.0) (Fig. S1). Under 0.8–1.0 mol L^–1^ NaCl conditions, the growth of all isolates was inhibited, and the growth of NRCB030, NRCB026, and NRCB010 was better that of the other isolates (Fig. S1).

### Bioremoval of NO_3_^–^ and NO_2_^–^ under saline conditions

**Fig. 2.**
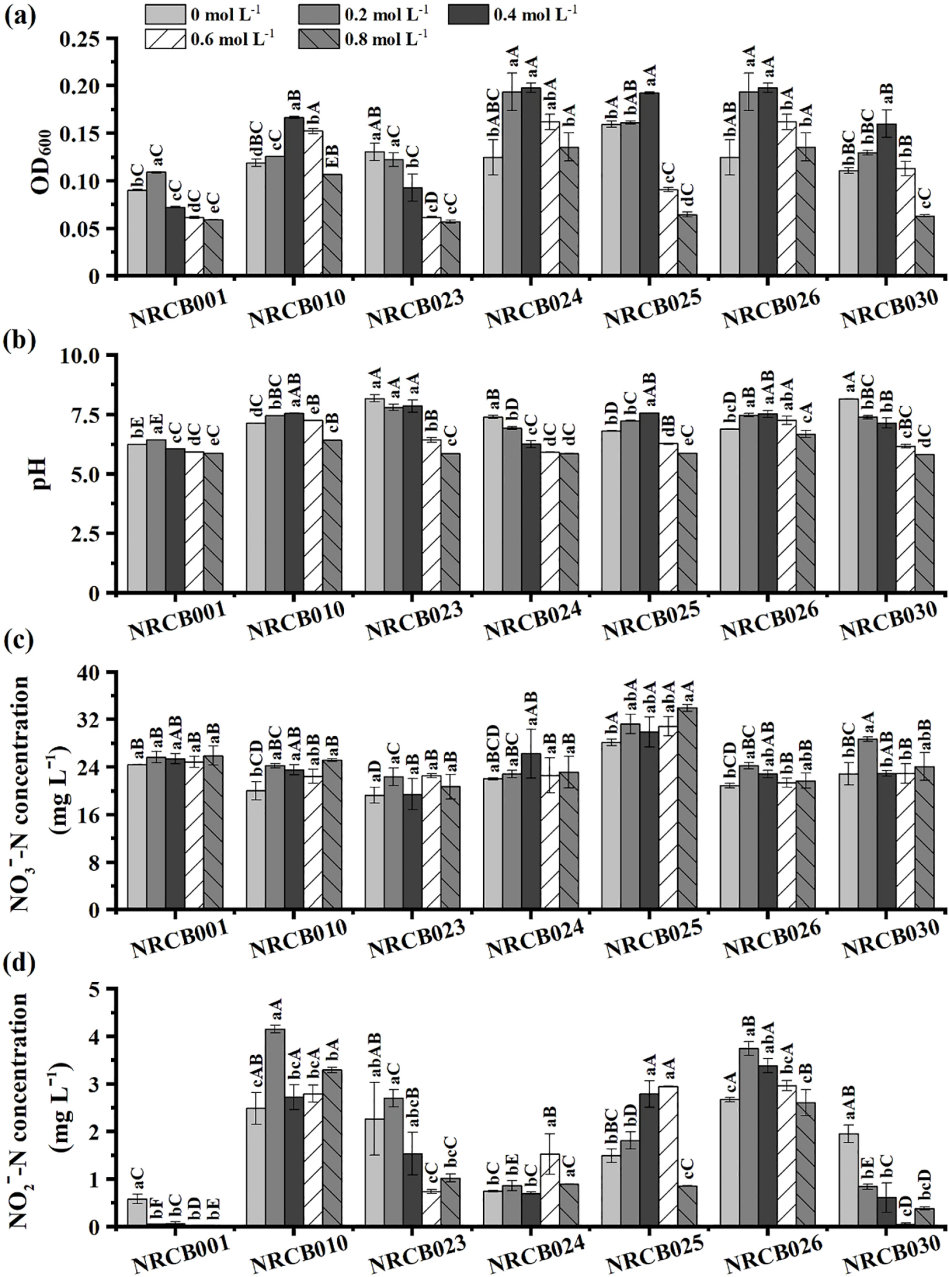
Nitrate and nitrite reduction by bacterial isolates in DM media with different NaCl concentrations. Data are means±standard errors (*n*=3). Data with different capital letters for different strains at the same NaCl concentration and lowercases for the same strain at different NaCl concentrations denote significant differences among treatments according to Duncan’s test (*P*<0.05).

The OD_600_ values of these isolates generally decreased when the concentration of NaCl was ≥0.4 mol L^–1^ (Fig. 2a), similar to pH values (Fig. 2b). pH values and NO_3_^–^-N and NO_2_^–^-N concentrations without the inoculation in DM media were not significantly affected (Fig. S2). NO_3_^–^-N removal rates were 71.9–80.7% under 0.4 mol L^–1^ NaCl conditions and ranged between 66.0–80.7% when the concentration of NaCl was 0.2–0.8 mol L^–1^ (Fig. 2c). NO_2_^–^-N concentrations in culture media markedly varied in each isolate and were lower than 5.0‍ ‍mg L^–1^ (Fig. 2d). NO_3_^–^-N concentrations remained almost constant, whereas NO_2_^–^-N concentrations generally decreased with an increase in salinity.

### Bacterial identification of NRCB010 and NRCB026

Two isolates, NRCB010 and NRCB026, were screened from the seven selected isolates in consideration of plant growth-promoting effects, salinity resistance, and N removal efficiency. The *16S rRNA* sequences of the two isolates were deposited in NCBI GenBank under accession numbers MZ165017 and MZ165016. Based on *16S rRNA* sequences, phylogenetic tree ana­lyses showed that NRCB010 belonged to *P. stutzeri* (Fig. S3a) and NRCB026 to *Bacillus* spp.. Based on MLSA, NRCB026 was further identified as *B. velezensis* (Fig. S3b).

### N removal by NRCB010 and NRCB026 in culture media with different initial pH and C sources

**Fig. 3.**
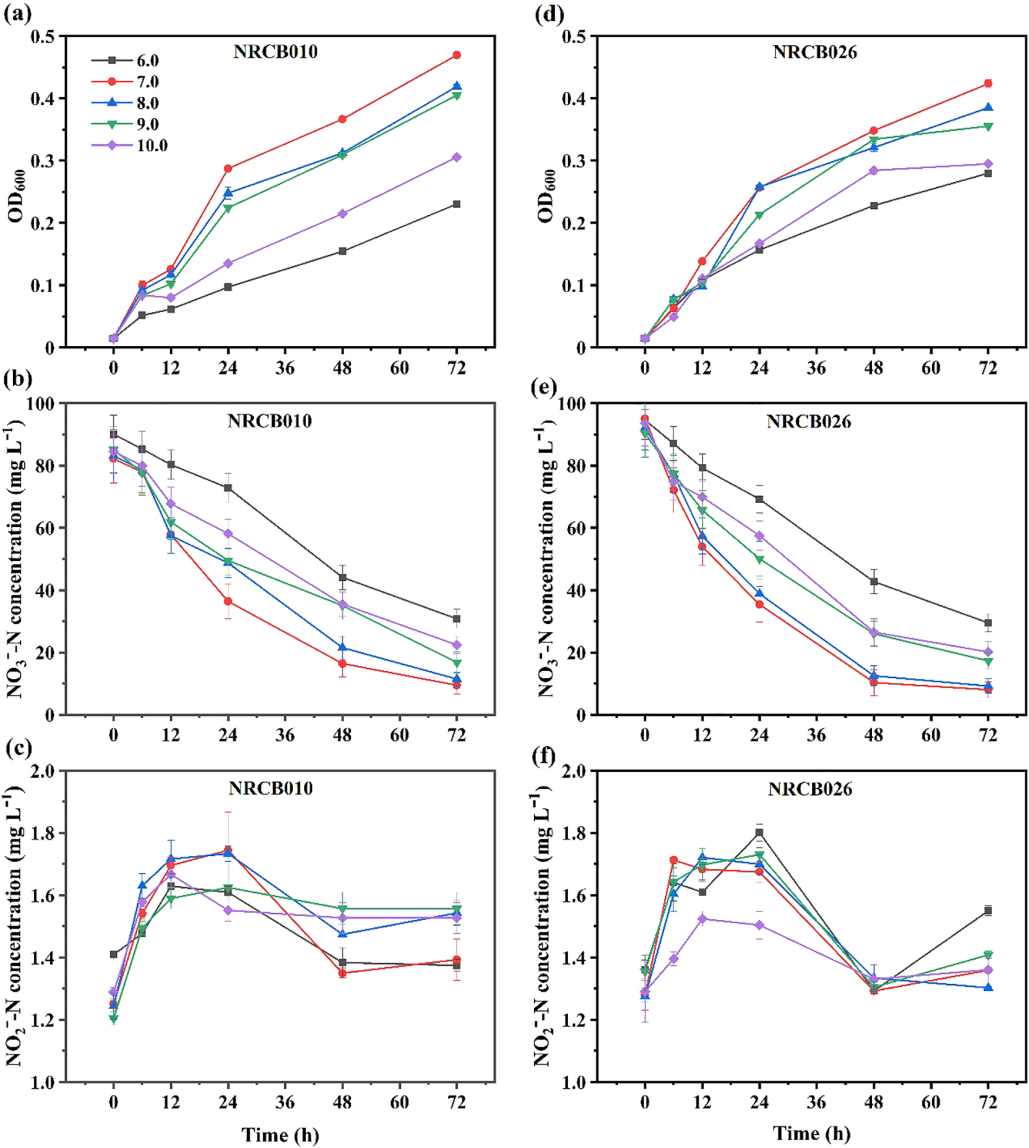
Nitrate and nitrite reduction by *Pseudomonas stutzeri* NRCB010 (a, b, and c) and *Bacillus velezensis* NRCB026 (d, e, and f) in DM media with different initial pH values. Data are means±standard errors (*n*=3).

The removal rates of NO_3_^–^-N and NO_2_^–^-N by NRCB010 and NRCB026 were evaluated at different initial pH of DM with the addition of 0.6 mol L^–1^ NaCl (Fig. 3). NO_3_^–^-N and NO_2_^–^-N concentrations without the inoculation in DM media at different initial pH were not significantly affected (Fig. S4). In general, the OD_600_ of these two strains exponentially increased with time. At pH>7.0, OD_600_ gradually decreased with an increase in pH (Fig. 3a and d). NO_3_^–^-N concentrations markedly decreased with time. At pH>7.0, OD_600_ gradually increased with an increase in pH (Fig. 3b and e). Maximum NO_3_^–^-N removal rates were achieved for NRCB010 (90.6%) and NRCB026 (92.0%) at an initial pH of 7.0 after a 72-h incubation. NO_2_^–^-N concentrations exponentially increased within ~12 h of the incubation, reaching a maximum after a 24-h incubation, and decreasing thereafter (Fig. 3c and f).

**Fig. 4.**
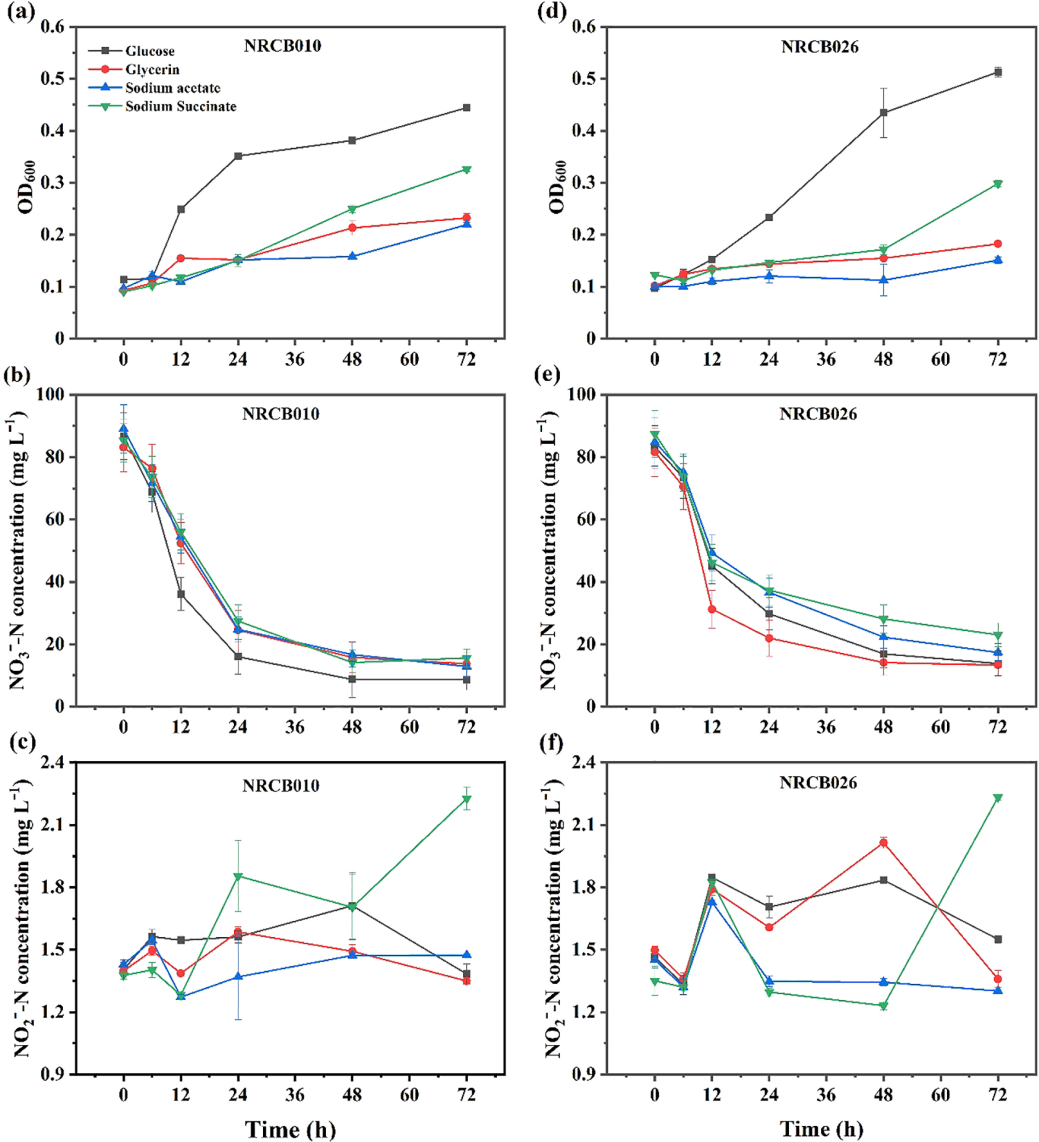
The microbial growth-promoting (OD_600_) and NO_3_^–^-N and NO_2_^–^-N removal abilities of *Pseudomonas stutzeri* NRCB010 (a, b, and c) and *Bacillus velezensis* NRCB026 (d, e, and f) in DM-based media supplemented with different C sources. Data are means±standard errors (*n*=3).

The removal rates of NO_3_^–^-N and NO_2_^–^-N by NRCB010 and NRCB026 were evaluated using different C sources in DM with the addition of 0.6 mol L^–1^ NaCl (Fig. 4). NO_3_^–^-N and NO_2_^–^-N concentrations without the inoculation in DM-based media with different C sources were not significantly affected (Fig. S5). When using glucose as the C source, the OD_600_ values of NRCB010 and NRCB026 were higher than those using glycerin, sodium acetate, or sodium succinate as the C source (Fig. 4a and c). NO_3_^–^-N concentrations markedly decreased with time after the inoculation, particularly within ~24 h of the incubation (Fig. 4b and e). NO_2_^–^-N concentrations ranged between 1.2 and 2.2‍ ‍mg L^–1^ after the inoculation with NRCB010 and NRCB026 (Fig. 4c and f).

### N removal from agricultural waters by NRCB010 and NRCB026

**Fig. 5.**
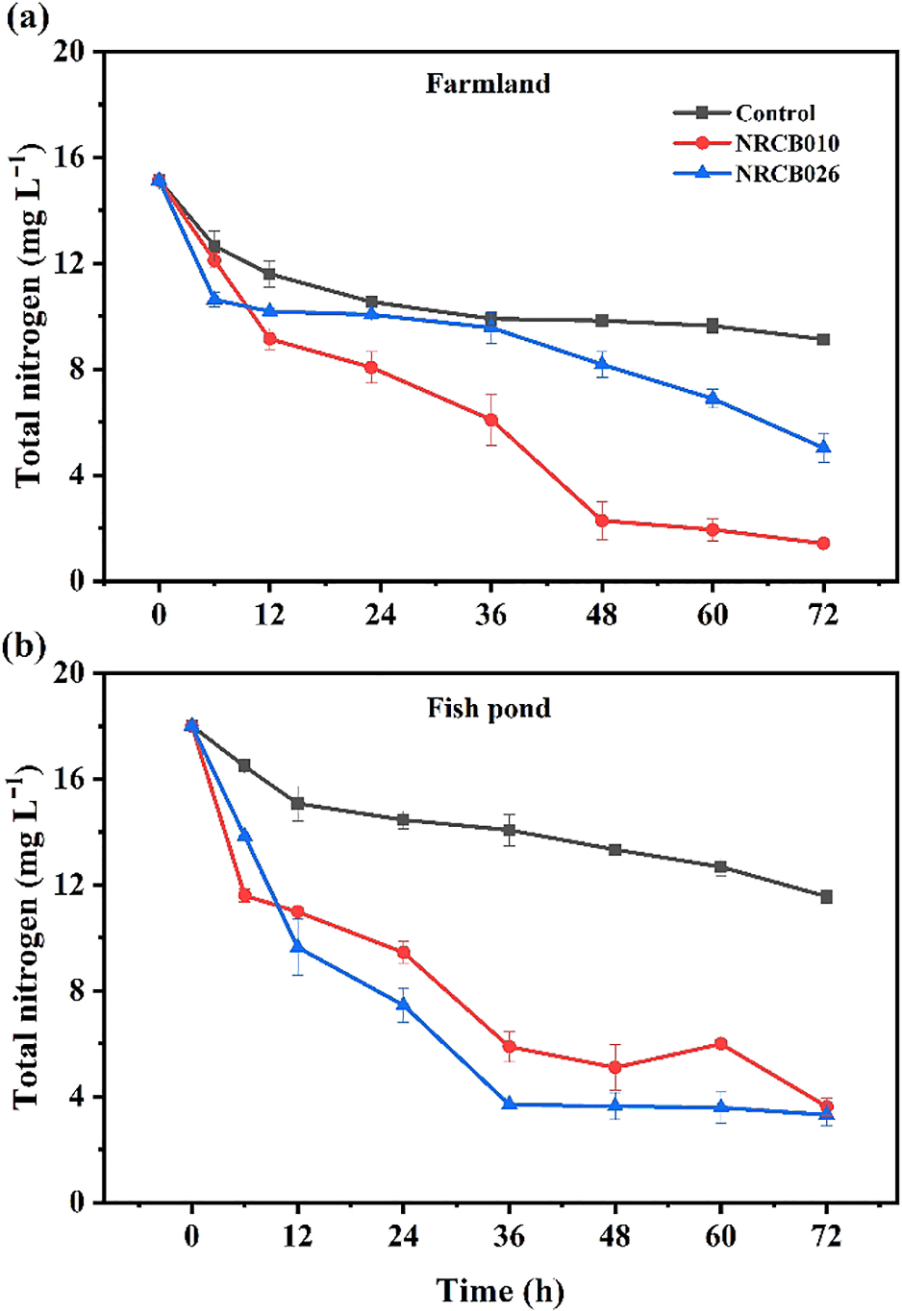
Nitrogen removal from farmland water (a) and fishpond water (b) by *Pseudomonas stutzeri* NRCB010 and *Bacillus velezensis* NRCB026. Values are means±standard errors (*n*=3).

The initial pH and TN concentration in the water sample collected from a farmland ditch were 7.1 and 15‍ ‍mg L^–1^, respectively. Without the inoculation, TN concentrations in non-inoculated farmland water and fishpond water decreased over time (Fig. 5). After a 72-h incubation with NRCB010 and NRCB026, TN concentrations decreased further to 1.4 and 5.0‍ ‍mg L^–1^ respectively (Fig. 5a). The TN removal rates of NRCB010 and NRCB026 were 90.6 and 66.7%, respectively. Moreover, for the water sample collected from a fishpond, the initial pH was 6.7 and the TN concentration was 18‍ ‍mg L^–1^. After 72 h of the incubation with NRCB010 and NRCB026, TN concentrations decreased to 3.6 and 3.3‍ ‍mg L^–1^, respectively (Fig. 5b). TN removal rates for the inoculated treatments of NRCB010 and NRCB026 were 79.9 and 81.6%, respectively.

## Discussion

PGPR play an important role in enhancing crop production and mitigating the adverse environmental effects of synthetic fertilizers, particularly N fertilizers (Zhang *et al.*, 2010; El-Sayed *et al.*, 2015; Gao *et al.*, 2016; Lu *et al.*, 2021). In the present study, *P. stutzeri* NRCB010 and *B. velezensis* NRCB026 dissolved phosphorus and produced siderophores and auxins, which significantly increased root lengths and the dry weights of tomato seedlings on agar plates. It currently remains unclear whether other mechanisms exist. The plant growth-promoting activities of the isolated bacterial strains also need to be evaluated in a pot experiment on tomato plants and several crops growth.

NRCB010 and NRCB026 exhibited promising N removal abilities (77.6–92.0%) at pH 7.0–10.0 and 3.5% salinity. The salinity levels of coastal aquaculture wastewater range between 2 and 35‍ ‍g L^–1^ NaCl (Li *et al.*, 2018). The salt-tolerant bacteria *Pannonibacter phragmitetus* F1 (Wang *et al.*, 2019), *Pseudomonas* sp. MSD4 (Zeng *et al.*, 2020), and *Bacillus* sp. N31 (Huang *et al.*, 2017) have been isolated and used for N removal from saline wastewater. The strains selected in the present study showed excellent salt resistance; moreover, three strains thrived at a salinity of 4.7%. NO_3_^–^-N removal rates at 3.5% salinity for NRCB010 and NRCB026 were 90.6 and 92.0%, respectively, at pH 7.0 and 88.5 and 82.7%, respectively, at pH 9.0. High salinity resistance and promising N removal by these two isolates indicate great potential for practical applications to agricultural wastewater, particularly in coastal or saline farmlands.

Inoculations with NRCB010 and NRCB026 enhanced N removal from two agricultural water samples. TN concentrations in non-inoculated farmland water and fishpond water decreased over time (Fig. 5). Non-inoculated control water may not have been sterilized and, thus, contained living microorganisms; some were denitrifiers that remove some N through denitrification. The *napA*, *norB*, and *nosZ* genes were successfully amplified from NRCB010 (Fig. S6a); therefore, NRCB010 may remove N from water via denitrification. Complete NO_3_^–^ removal to dinitrogen gas (denitrification) generally involves a series of reducing enzymatic reactions of microbes under anaerobic conditions; however, NO_3_^–^ removal tests were performed under aerobic conditions in the present study. Additional experiments need to be conducted to confirm that the strains are aerobic denitrifiers, such as an oxygen sensitivity test on the transcription of denitrification functional genes. Since only *nirS* was detected in NRCB026 using the current primers (Fig. S3b), PCR detection did not appear to be sufficient to confirm the existence of other denitrification functional genes or NRCN026 may have removed N by the assimilation of NO_3_^–^ into bacterial cells.

These strains may be invaluable microbial resources for the development of novel biofertilizers with NO_3_^–^-N removal abilities. Consequently, they may decrease synthetic fertilizer input, enhance crop production, and decrease NO_3_^–^-N concentrations in farmland drainage water, thereby reducing agricultural nonpoint source pollution. Furthermore, they may reduce NO_3_^–^ pollution in the groundwater by decreasing NO_3_^–^-N concentrations in soil leachates. The efficient use of N after the application of biofertilizers with these two strains warrants further investigation.

In conclusion, seven bacterial strains that exert plant growth-promoting effects and exhibit NO_3_^–^-N removal abilities were isolated from plant rhizosphere soil. Under non-salinity conditions, NO_3_^–^-N removal rates were 71.9–80.7%; at 1.2–3.5% salinity, NO_3_^–^-N removal rates were 66.0–80.7%. Of these seven isolates, NRCB010 and NRCB026 exerted stronger plant growth-promoting effects, salinity resistance, and NO_3_^–^-N removal abilities. These two isolates had N removal rates of 77.6–92.0% at pH 7.0–10.0 and 3.5% salinity. Moreover, maximum NO_3_^–^-N removal rates at pH 7.0 were 90.6 and 92.0% for NRCB010 and NRCB026, respectively. Higher NO_3_^–^-N removal rates were achieved with glucose or glycerin as the C source than with sodium acetate and sodium succinate. After the inoculation with NRCB010 and NRCB026, TN removal rates were 90.6 and 66.7%, respectively, in farmland effluents and 79.9 and 81.6%, respectively, in aquaculture water. Overall, they may be used for the development of novel biofertilizers or to reduce N pollution in water.

## Citation

Zhang, H., Shen, W., Ma, C., Li, S., Chen, J., Mou, X., et al. (2022) Simultaneous Nitrogen Removal and Plant Growth Promotion Using Salt-‍tolerant Denitrifying Bacteria in Agricultural Wastewater. *Microbes Environ ***37**: ME22025.

https://doi.org/10.1264/jsme2.ME22025

## Supplementary Material

Supplementary Material

## Figures and Tables

**Table 1.** Plant growth-promoting characteristics of bacterial isolates.

Isolate	IAA production (μg mL^–1^)	Dissolved phosphorus (mg L^–1^)	Siderophore (%)	ACC deaminase activity
NRCB001	1.19	59.62	5.85	positive
NRCB010	33.33	51.35	34.04	positive
NRCB023	25.38	56.37	29.08	positive
NRCB024	18.48	51.72	52.30	positive
NRCB025	15.83	56.42	44.06	positive
NRCB026	19.06	51.95	32.27	positive
NRCB030	1.84	52.38	19.24	positive
