# Supplementary material for: Simultaneous Nitrogen Removal and Plant Growth Promotion Using Salt-‍tolerant Denitrifying Bacteria in Agricultural Wastewater
Source: Microbes Environ. 2022 Sep 17;37(3):ME22025. doi: 10.1264/jsme2.ME22025 (PMC9530716; doi:10.1264/jsme2.ME22025)
Supplement: Supplementary file 1 — Supplementary Material [file 37_22025_s1.pdf]

**Supplementary Materials and Methods**

*Characterization of bacterial denitrifying genes of NRCB010 and NRCB026*

Ammonia monooxygenase gene (*amoA*), periplasmic nitrate reductase gene (*napA*), nitrite reductase gene (*nirS*), nitric reductase gene (*norB*), and nitrous oxide reductase gene (*nosZ*) were amplified using an S1000 Thermal Cycler (Bio-Rad, USA) in 10 μL reaction volumes containing 5 μL of 2×Taq Master Mix (Dye Plus), 200 ng template DNA, 1 μL of each primer (10 μmol $L^{-1}$), and sterile distilled water. The primers and thermal profiles were as listed in Table S1.

**Table S1.** Primers and thermal profiles employed for PCR amplification of target sequences.

| Target gene | Primer name | Primer sequence（5’ to 3’） | Thermal profile | Product size (bp) | Reference |
|---|---|---|---|---|---|
| *16S rRNA* | 27F | AGAGTTTGATCMTGGCTCAG | ① 94°C, 10 min | 1466 | Hongoh et al., 2003 |
| | 1492R | GGTTACCTTGTTACGACTT | ② 94°C, 30 s | | |
| *gyr A* | gyrA-285 | TGTCAAAAATCGCAATGGAA | ③ 55°C, 30 s | 1597 | This manuscript |
| | gyrA-1881 | TGTTTCTTGCCGTCAGTCAG | ④ 72°C, 90 s | | |
| *gyr B* | gyrB-66 | GACCACTCTTGACGTTACGGT | ⑤ GO TO ②, 30 cycles | 997 | This manuscript |
| | gyrB-1062 | TCTGGCTTTCTCAACGTTCAGA | ⑥ 72°C, 7 min | | |
| *amoA* | AMO-F | GGGAATTCAGAAATCCTGAAAGCGGC | ① 94°C, 10 min | 665 | Sinigalliano et al., 1995 |
| | AMO-R | GGGGATCCGATACGAACGCAGAGAAG | ② 94°C, 60 s | | |
| | | | ③ 50°C, 30 s | | |
| | | | ④ 72°C, 30 s | | |
| | | | ⑤ GO TO ②, 30 cycles | | |
| | | | ⑥ 72°C, 10 min | | |
| *napA* | napA-F1188 | TCTGGACCATGGGCTTCAACCA | ① 94°C, 10 min | 884 | Chen et al., 2012 |
| | napA-R2064 | ACGACGACCGGCCAGCGCAG | ② 94°C, 60 s | | |
| *nirS* | nirS-1F | CCTA(C/T)TGGCCGCC(A/G)CA(A/G)T | ③ 56°C, 60 s | 975 | Braker et al., 1998 |
| | nirS-6R | CGTTGAACTT(A/G)CCGGT | ④ 72°C, 90 s | | |
| | | | ⑤ GO TO ②, 30 cycles | | |
| | | | ⑥ 72°C, 10 min | | |
| *norB* | norB-F0755 | TGCTGTTCCGTCTGGAGAA | ① 94°C, 5 min | 991 | Zhao et al., 2018 |
| | norB-R1648 | CGTAGCGACCTTCATAGAGG | ② 94°C, 30 s | | |
| | | | ③ 57°C, 30 s | | |
| | | | ④ 72°C, 45 s | | |
| | | | ⑤ GO TO ②, 30 cycles | | |
| | | | ⑥ 72°C, 7 min | | |
| *nosZ* | nosZ-F1181 | CGCTGTTCITCGACAGYCAG | ① 95°C, 10 min | 700 | Rich et al., 2003 |
| | nosZ-R1880 | ATGTGCAKIGCRTGGCAGAA | ② 95°C, 30 s | | |
| | | | ③ 55°C, 30 s | | |
| | | | ④ 72°C, 45 s | | |
| | | | ⑤ GO TO ②, 32 Cycles | | |
| | | | ⑥ 72°C, 7 min | | |

**Table S2.** Physiological and biochemical characteristics of the bacteria.

| Strain | Species | Isolated from | Ammonia production test | Nitrate reduction test | Starch hydrolysis test | V-P test | pH 6 | pH 5 | Glycerin |
|---|---|---|---|---|---|---|---|---|---|
| NRCB001 | *Bacillus megaterium* | Yixing, China | + | − | + | − | + | − | + |
| NRCB010 | *Pseudomonas stutzeri* | Yixing, China | + | + | + | − | + | − | + |
| NRCB023 | *Pseudomonas stutzeri* | Yixing, China | + | + | + | − | + | − | + |
| NRCB024 | *Pseudomonas stutzeri* | Yixing, China | + | + | + | + | + | − | + |
| NRCB025 | *Pseudomonas stutzeri* | Yixing, China | + | + | + | − | + | − | + |
| NRCB026 | *Bacillus velezensis* | Huizhou, China | − | + | − | + | + | + | + |
| NRCB030 | *Achromobacter denitrificans* | Nanjing, China | − | + | − | − | + | − | − |

"+" and "−" indicate positive and negative, respectively.

**Fig. S1.** The microbial growth (OD600) of candidate strains cultured in LB media with different NaCl concentrations. Data are means ± standard error (*n* = 4). Data with different capital letters for different strains at the same NaCl concentration and lowercases for the same strain at the different NaCl concentrations denote significant differences among treatments according to Duncan's test ($P<0.05$).

**Fig. S2.** Nitrate and nitrite concentration without inoculation in DM media with different NaCl concentrations. Data are means ± standard error (*n* = 3). Data with same lowercases denote no significant difference at the different NaCl concentrations according to Duncan's test ($P<0.05$).

**Fig. S3.** Neighbor-joining phylogenetic analysis of *Pseudomonas stutzeri* NRCB010 based on *16S rRNA* sequences (a) and *Bacillus velezensis* NRCB026 based on multilocus (*gyr A* and *gyr B*) sequence analysis (b) constructed in MEGA 5.0 with 1000 bootstrap replicates.

**Fig. S4.** Nitrate and nitrite concentration without inoculation in DM media with different initial pH values. Data are means ± standard error (*n* = 3).

**Fig. S5.** Nitrate and nitrite concentration without inoculation in DM-based media supplemented with different C-sources. Data are means ± standard error (*n* = 3).

**Fig. S6.** Nitrogen metabolism related gene amplification of *Pseudomonas stutzeri* NRCB010 (a) and *Bacillus velezensis* NRCB026 (b). Lane 1, DL 2000, DNA marker; lane 2, *amoA*; lane 3, *napA*; lane 4, *nirS*; lane 5, *norB*; lane 6, *nosZ*.

**Fig. S1.**

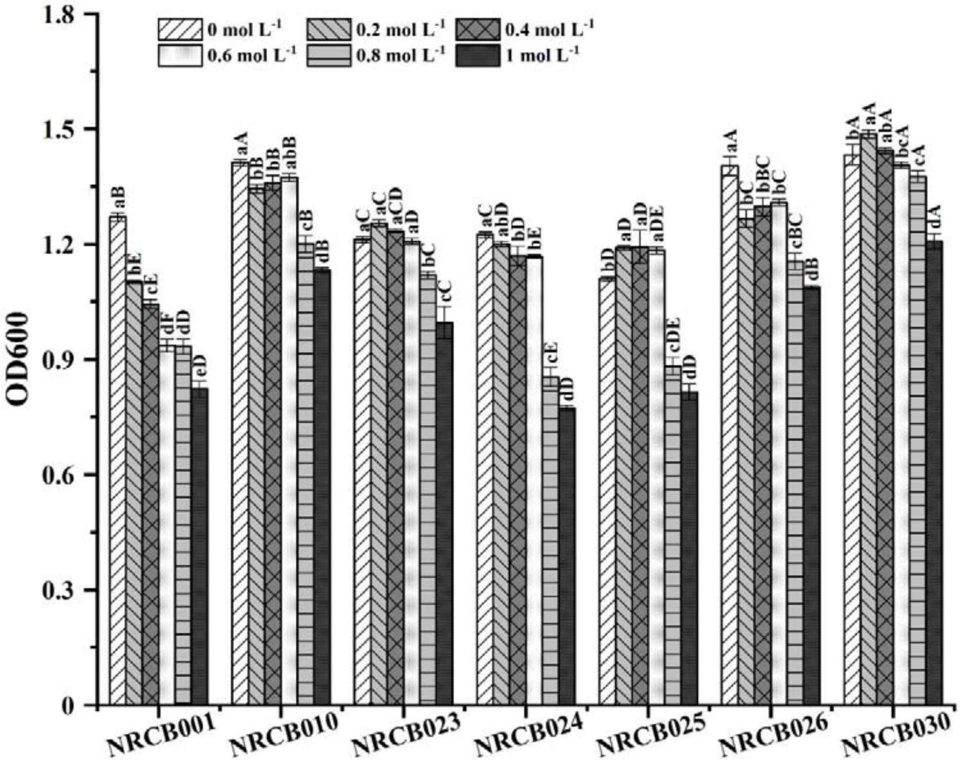
0 mol L-1
0.2 mol L-1
0.4 mol L-1
0.6 mol L-1
0.8 mol L-1
1 mol L-1
OD600
1.8
1.5
1.2
0.9
0.6
0.3
0
NRCB001
NRCB010
NRCB023
NRCB024
NRCB025
NRCB026
NRCB030

**Fig. S2.**

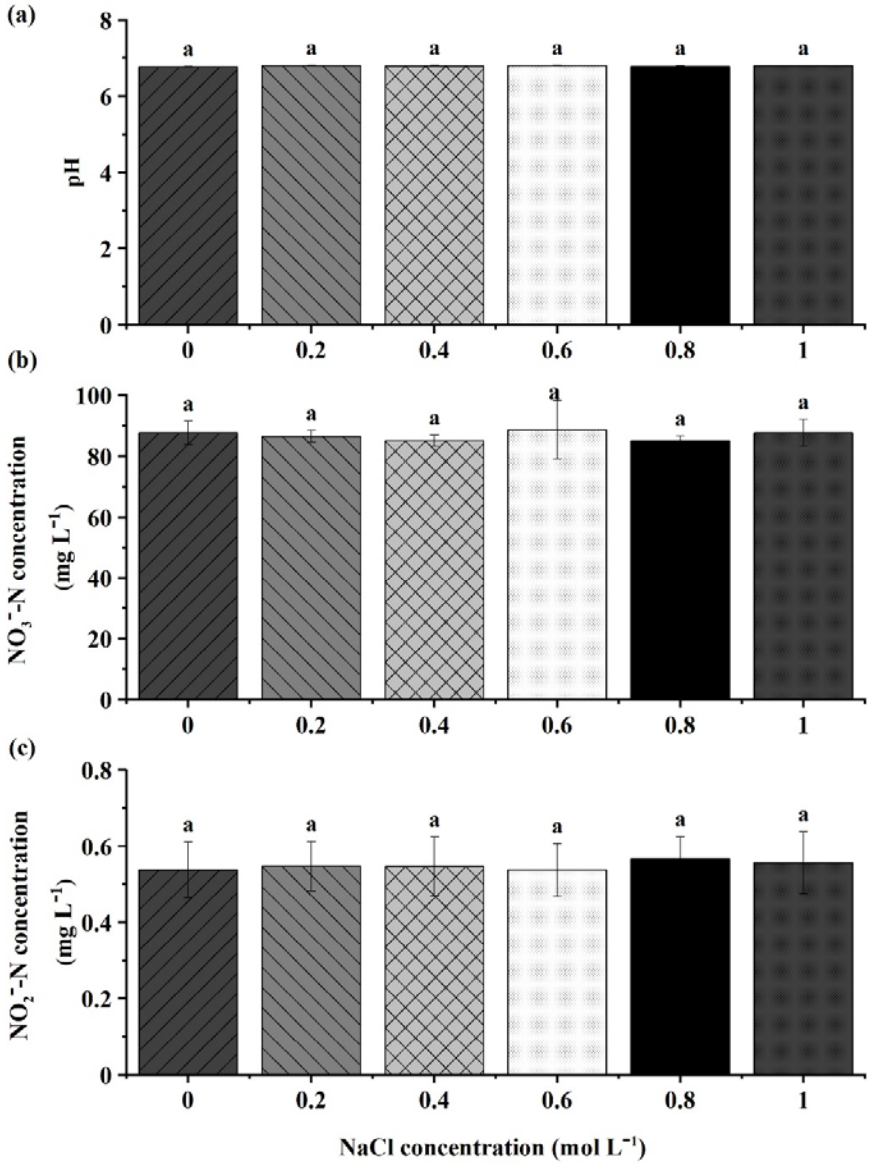
(a)
pH
8
6
4
2
0
a
0
0.2
0.4
0.6
0.8
1
(b)
NO3⁻-N concentration (mg L⁻¹)
100
80
60
40
20
0
(c)
NO2⁻-N concentration (mg L⁻¹)
0.8
0.6
0.4
0.2
NaCl concentration (mol L⁻¹)

**Fig. S3.**

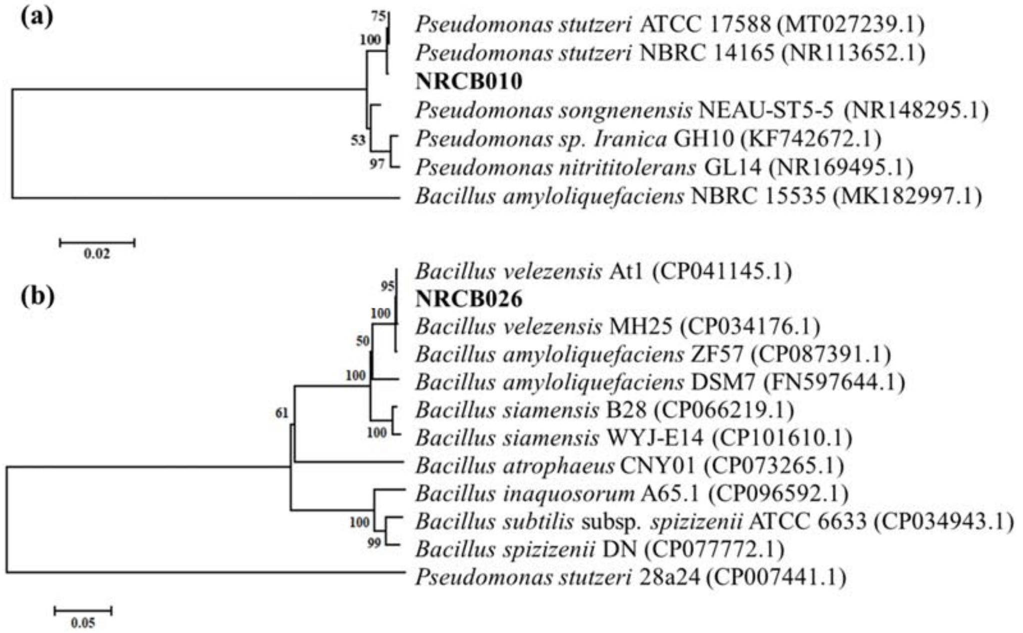

(a)
75
100
Pseudomonas stutzeri ATCC 17588 (MT027239.1)
Pseudomonas stutzeri NBRC 14165 (NR113652.1)
NRCB010
Pseudomonas songnenensis NEAU-ST5-5 (NR148295.1)
53
Pseudomonas sp. Iranica GH10 (KF742672.1)
97
Pseudomonas nitrititolerans GL14 (NR169495.1)
Bacillus amyloliquefaciens NBRC 15535 (MK182997.1)
0.02
(b)
Bacillus velezensis At1 (CP041145.1)
95
NRCB026
100
Bacillus velezensis MH25 (CP034176.1)
50
Bacillus amyloliquefaciens ZF57 (CP087391.1)
100
Bacillus amyloliquefaciens DSM7 (FN597644.1)
61
Bacillus siamensis B28 (CP066219.1)
100
Bacillus siamensis WYJ-E14 (CP101610.1)
Bacillus atrophaeus CNY01 (CP073265.1)
Bacillus inaquosorum A65.1 (CP096592.1)
100
Bacillus subtilis subsp. spizizenii ATCC 6633 (CP034943.1)
99
Bacillus spizizenii DN (CP077772.1)
Pseudomonas stutzeri 28a24 (CP007441.1)
0.05

**Fig. S4.**

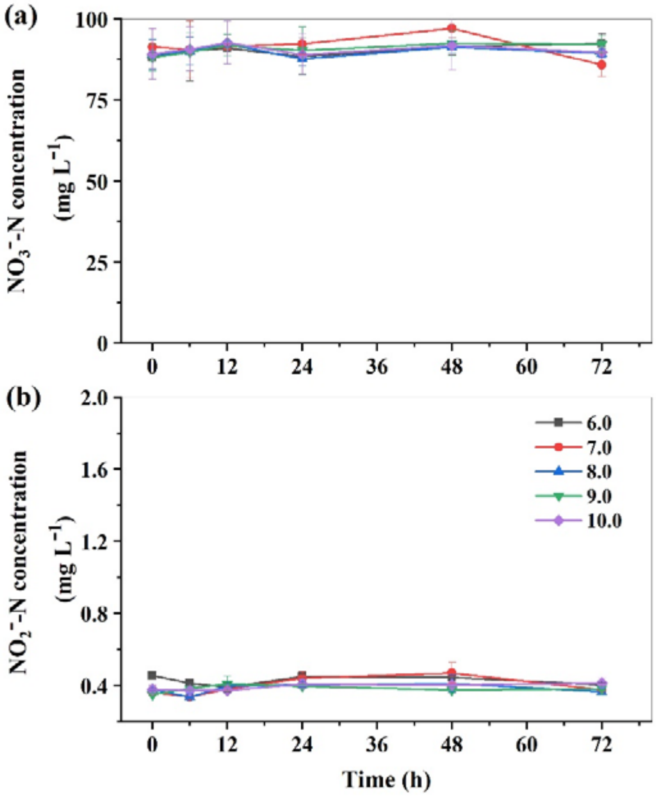
(a)
$NO_3^-$-N concentration (mg $L^{-1}$)
0
25
50
75
100
0
12
24
36
48
60
72
(b)
$NO_2^-$-N concentration (mg $L^{-1}$)
0.4
0.8
1.2
1.6
2.0
6.0
7.0
8.0
9.0
10.0
Time (h)

**Fig. S5.**

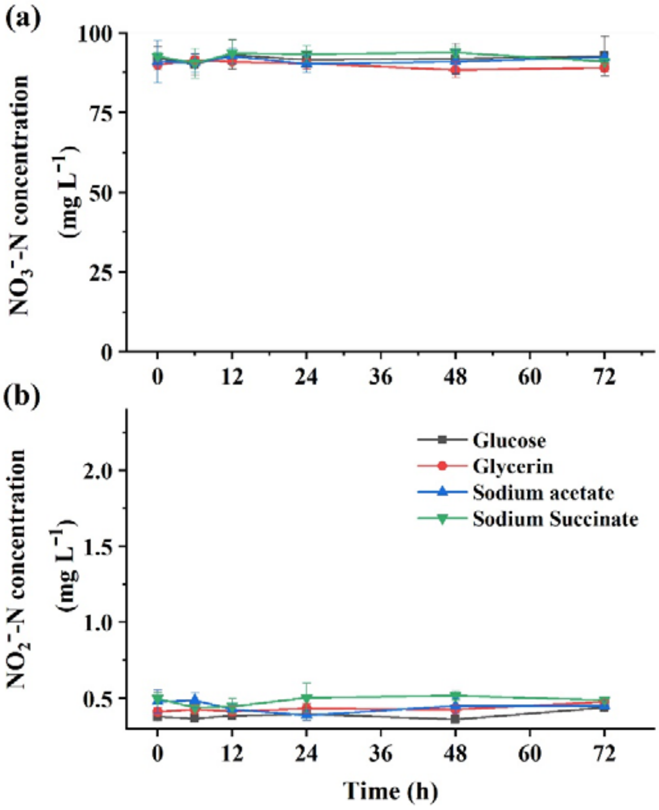

(a)
$NO_3^-$-N concentration (mg L$^{-1}$)
100
75
50
25
0
0
12
24
36
48
60
72
(b)
$NO_2^-$-N concentration (mg L$^{-1}$)
2.0
1.5
1.0
0.5
Glucose
Glycerin
Sodium acetate
Sodium Succinate
0
12
24
36
48
60
72
Time (h)

**Fig. S6.**

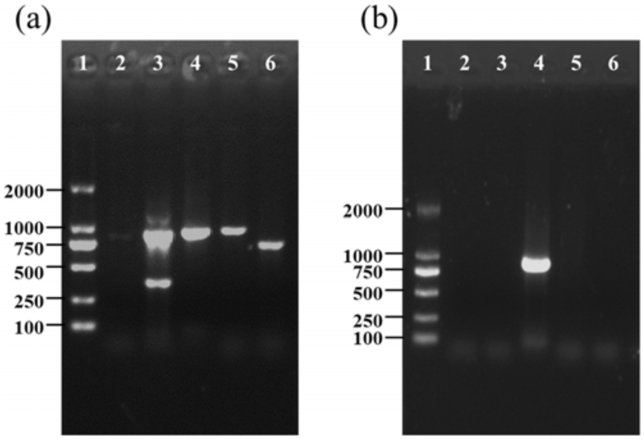
(a)
1 2 3 4 5 6
2000
1000
750
500
250
100
(b)
1 2 3 4 5 6
2000
1000
750
500
250
100

**Supplementary References**


Braker, G., Fesefeldt, A., and Witzel, K.P. (1998) Development of PCR primer systems for amplification of nitrite reductase genes (*nirK* and *nirS*) to detect denitrifying bacteria in environmental samples. *Appl Environ Microbiol* **64**: 3769-3775.

Chen, Q., and Ni, J.R. (2012) Ammonium removal by *Agrobacterium sp* LAD9 capable of heterotrophic nitrification-aerobic denitrification. *J Biosci Bioeng* **113**: 619-623.

Hongoh, Y., Ohkuma, M., and Kudo, T. (2003) Molecular analysis of bacterial microbiota in the gut of the termite *Reticulitermes speratus* (Isoptera; Rhinotermitidae). *Fems Microbiol Ecol* **44**: 231-242.

Rich, J.J., Heichen, R.S., Bottomley, P.J., Cromack, Jr.K., and Myrold, D.D. (2003) Community composition and functioning of denitrifying bacteria from adjacent meadow and forest soils. *Appl Environ Microbiol* **69**: 5974-5982.

Sinigalliano, C.D., Kuhn, D.N., and Jones, R.D. (1995) Amplification of the *amoA* gene from diverse species of ammonium-oxidizing bacteria and from an indigenous bacterial population from seawater. *Appl Environ Microbiol* **61**: 2702-2706.

Zhao, B., Cheng, D.Y., Tan, P., An, Q., and Guo, J.S. (2018) Characterization of an aerobic denitrifier *Pseudomonas stutzeri* strain XL-2 to achieve efficient nitrate removal. *Bioresour Technol* **250**: 564-573.
